# Supplementary material for: Complexome profiling of the Chlamydomonas psb28 mutant reveals TEF5 as an early PSII assembly factor
Source: Plant Cell. 2025 Mar 17;37(6):koaf055. doi: 10.1093/plcell/koaf055 (PMC12164590; doi:10.1093/plcell/koaf055)
Supplement: koaf055_Supplementary_Data [file koaf055_supplementary_data.zip › psb28_interactive.zip!/plots/intensity/qualityPlots/peptides/RPA1_Cre12.g529600.t1.2.html]

### Cre12.g529600.t1.2

Trivial name: RPA1  
SeqCoverage [median]: 1.80  
Mass: 203.86 kDa  
  
 

| Sequence | MissedCleavage | Mass [Da] | wtIntensity | muIntensity | Score |
| --- | --- | --- | --- | --- | --- |
| DKKGAKGSAGEGLPAELLADPASHAR | 3 | 2545.32 | 34.14 | 0.00 | 36.58 |
| KAGVGIGK | 1 | 728.45 | 564.72 | 1551.56 | 73.98 |
|  |  |  | 598.9 | 1551.6 |  |
